# Mental Ill-Health in young people with systemic autoinflammatory disease – a scoping review

**DOI:** 10.1007/s00296-025-05864-w

**Published:** 2025-04-18

**Authors:** Amanda Clarke, Caitlin McDowell, Paul Badcock

**Affiliations:** 1 Centre for Youth Mental Health, The University of Melbourne, Victoria Parkville, Australia; 2 Orygen, Victoria Parkville, Australia

**Keywords:** Systemic autoinflammatory disease, Mental disorder, Depression, Anxiety, Young person, Inflammation

## Abstract

Systemic autoinflammatory disorders (SAID), immune dysregulation disorders with onset frequently occurring in youth, are defined by unprovoked inflammation. Research suggest inflammation is associated with the pathogenesis of mental ill-health. Separately, research has identified that mental ill-health is prevalent in people with immune dysregulation disorders compared to healthy controls or those with other chronic diseases, and the psychosocial impacts of these diseases on quality of life can be debilitating. This review aimed to broadly identify the extent and nature of research involving young people with SAIDs and mental ill-health. A scoping review was conducted across 6 databases of peer-reviewed articles referring to SAIDs and mental ill-health in young people. Of 727 studies, 41 met the inclusion criteria. Thirty-seven were observational studies, including 18 case studies or series. Four studies were treatment trials. Fourteen observational studies investigated the prevalence of mental ill-health in a SAID population with large sample sizes. Most studies were conducted in Türkiye, involving Behçet’s syndrome or familial Mediterranean fever and anxiety, depression, and psychosis were strongly represented. Findings suggest an association between mental ill-health and SAID activity in young people. Anxiety and depression were associated with some specific SAIDs duration, symptom severity, SAID flare recency and frequency, and SAID treatment effects. Co-occurrence and resolution of SAID flares and mental ill-health with treatment were frequent themes in case studies of some SAIDs. Further research is required on the prevalence and risk of developing mental ill-health among young people with SAIDs, along with associations between mental ill-health, other SAIDs and disease activity, other participant variables, and appropriate management of mental ill-health in this population.

**Supplementary Information:**

The online version contains supplementary material available at 10.1007/s00296-025-05864-w.

## Introduction

Systemic autoinflammatory diseases (SAIDs) are disorders of the innate immune system defined by unprovoked episodes of inflammation [1], with onset predominantly occurring in adolescence [2]. The role of inflammation in the pathogenesis of mental ill-health has gained increasing attention in recent decades, with multiple lines of evidence supporting this association [3]. In parallel, research outlining the high prevalence and bidirectional association between disorders of the immune system and mental ill-health has emerged [4–7]. Consistent with these findings, some research demonstrates the high prevalence of mental ill-health among people with SAIDs [8], however little is known about this association in young people [9]. This is particularly concerning given the high rates of SAID onset in adolescence [2], and the impact and prevalence of mental ill-health in young people generally [10], with SAIDs and mental ill-health both being associated with significant morbidity and lower quality of life [11–13].

### Inflammation, immune dysregulation and mental health disorders

The immune system maintains homeostasis via its two arms: the broad nonspecific response of the innate immune system and a later targeted response of the adaptive immune system [14]. Inflammation is a key component of innate immune system activity, involving complex cascading chemical processes with key actors being inflammatory cytokines [15]. Pro-inflammatory cytokines, triggered by damaged tissue or invading pathogens, may enter the central nervous system and manifest acutely as sickness behaviours, including lethargy, reduced activity and cognitive impairment [16]. Importantly, pathological immune function in the form of chronic or recurrent acute inflammation may contribute to mental ill-health [17].

Multiple systematic reviews and meta analyses have confirmed that inflammation, measured as high levels of pro-inflammatory cytokines, occurs in adults [18–21] and young people [22–24] with a range of mental disorders, and that it declines with both mental health treatment and recovery [25, 26]. Early life inflammation associated with childhood adversity and infection has also been linked to increased risk of mental ill-health later in life [27–29].

Research also suggests an association between mental ill-health and immune dysregulation disorders that arise from under or overactivation of either arm of the immune system [30, 31]. Young people with such disorders experience increased rates of depression, anxiety, schizophrenia, eating disorders, obsessive compulsive disorder, tic disorders, somatic complaints, and behavioral disorders compared to healthy young people or those with other serious chronic illness [7, 32–34]. Less is known however, about mental ill-health in people with SAIDs, particularly young people.

### Systemic autoinflammation and mental ill-health

SAIDs are a recently defined family of immune dysregulation disorders driven by unprovoked recurrent or continuous inflammation [35]. Hallmark symptoms of SAID activity are raised serum inflammatory markers, fever and a wide range of physical manifestations involving many body systems [36]. Since the identification of the first gene causing SAID in 1997, more than forty monogenic SAIDs have been identified [2] and a further 40–60% of identified SAID cases remain clinically undifferentiated [36]. SAIDs are very rare diseases with a prevalence as low as 1:1,000,000, depending on disease and locality [36]. Disease rarity, limited clinical experience and community awareness, and evolving disease classifications [37] impede clinical and research progress.

Mental health symptoms are not included in any SAID classification system [36], although some research suggests that these may be integral to SAID activity [40]. It is more generally assumed that mental ill-health is secondary to, or comorbid with, disease manifestation [41]. Mental ill-health has been investigated in adults and children with particular SAIDs, such as Behçet’s syndrome [38] and familial Mediterranean fever (FMF) [39], and the psychosocial impacts of the diseases on functional capacity and quality of life are significant [12]. However, research on mental ill-health in young people with SAIDs is limited [9], and to our knowledge, no systematic or scoping reviews have been conducted to date. Importantly, young people are, in general, more likely to experience mental ill-health than other age groups [10]. The highest burden of disease due to mental ill-health at any time of life occurs during youth [13]. Risk factors for [43], and the prevalence of [44], psychiatric disorders are cumulative and young people with chronic disease are at additional risk [32, 45]. Limited understanding of the nature of the association between SAID activity and mental ill-health may lead to gaps in the recognition, assessment, diagnosis and management of psychiatric illness in this population [42]. Given the findings across multiple lines of research outlined above, there are clear indications that young people with SAIDs are at increased risk of developing mental ill-health. Furthermore, the mental ill-health that young people with SAIDs experience may in part be associated with disease activity and inflammation, but these associations have yet to be investigated.

Scoping reviews aim to broadly examine heterogeneous topics to identify novel patterns of association that may indicate new lines of investigation [46]. Although SAIDs are a heterogenous family, a commonality among all of these conditions is recurrent non-infectious acute and chronic inflammation impacting many systems, subsequent mental and behavioural processes, and indirectly, social systems, over time. Homogeneity is also elusive in mental health, where different causal pathways lead to the same mental disorder and comorbidity is the norm [47]. This is most evident in youth, where distress states and sub-threshold non-specific symptoms can regularly progress to anxiety, mood, somatic and/or psychotic syndromes. A broad and holistic review investigating these two heterogeneous, but seemingly related, variables is clearly needed. Accordingly, the aim of this scoping review is to map research related to SAIDs and mental ill-health in young people, particularly the association between mental ill-health, SAID activity and treatment. Study characteristics will be explored, including study design, location, sample size and age [48]. This review will consolidate evidence and identify gaps in the literature to inform future research and practice.

## Methods

Scoping reviews aim to map the extent, range and nature of research activity and evidence in a given area [49]. The authors developed a scoping review protocol to explore evidence surrounding the relationship between SAIDs and mental ill-health based on the JBI Manual of Evidence Synthesis methods [50], using the Preferred Reporting Items for Systematic Reviews and Meta-Analyses (PRISMA-ScR) guidelines for scoping reviews [46]. The scoping review was conducted between July 2023 and January 2025.

### Search strategy

For inclusion in the review, studies needed to focus on young people with at least one SAID and refer explicitly to the occurrence of mental ill-health. SAIDs were identified via various collective nomenclature, such as “periodic fever syndromes”, or via a specific SAID, as defined by Gutierrez and Lapidus [2], Gattorno et al. [37], Hausmann et al. [42] and/or Krainer et al. [36]. All mental disorders were included, including generic diagnoses such as anxiety, depression and psychosis. Studies reporting cognitive, emotional, or behavioural symptoms of mental ill-health were also eligible for inclusion. Neurological symptoms, cognitive impairment, and global developmental syndromes were excluded when no mental ill-health was reported. Studies published on or after 1997 were included, as this was the year the first genetically classified SAID was identified, leading to the definition of the SAID disease family [36]. Studies involving mixed SAID and non-SAID conditions were included where SAID specific data were reported separately. Young people were defined as aged 12 to 29 years [51] and studies were included if the mean or median age of participants was within this age range or data relating to the age group were analysed separately. All study settings and geographic locations were included. Only peer-reviewed primary research articles written in English were included. An initial search was conducted using six electronic databases: Medline, Emcare, PsycINFO, Embase, CINAHL and Scopus. Search terms were related to young people, SAIDs and mental ill-health or symptoms. MeSH terms were used for mental disorders and for autoinflammatory diseases where relevant, in addition to terms identified in the literature. See Supplemental material for full search criteria. Reference lists of included articles were also searched.

### Study selection

**Fig. 1:**
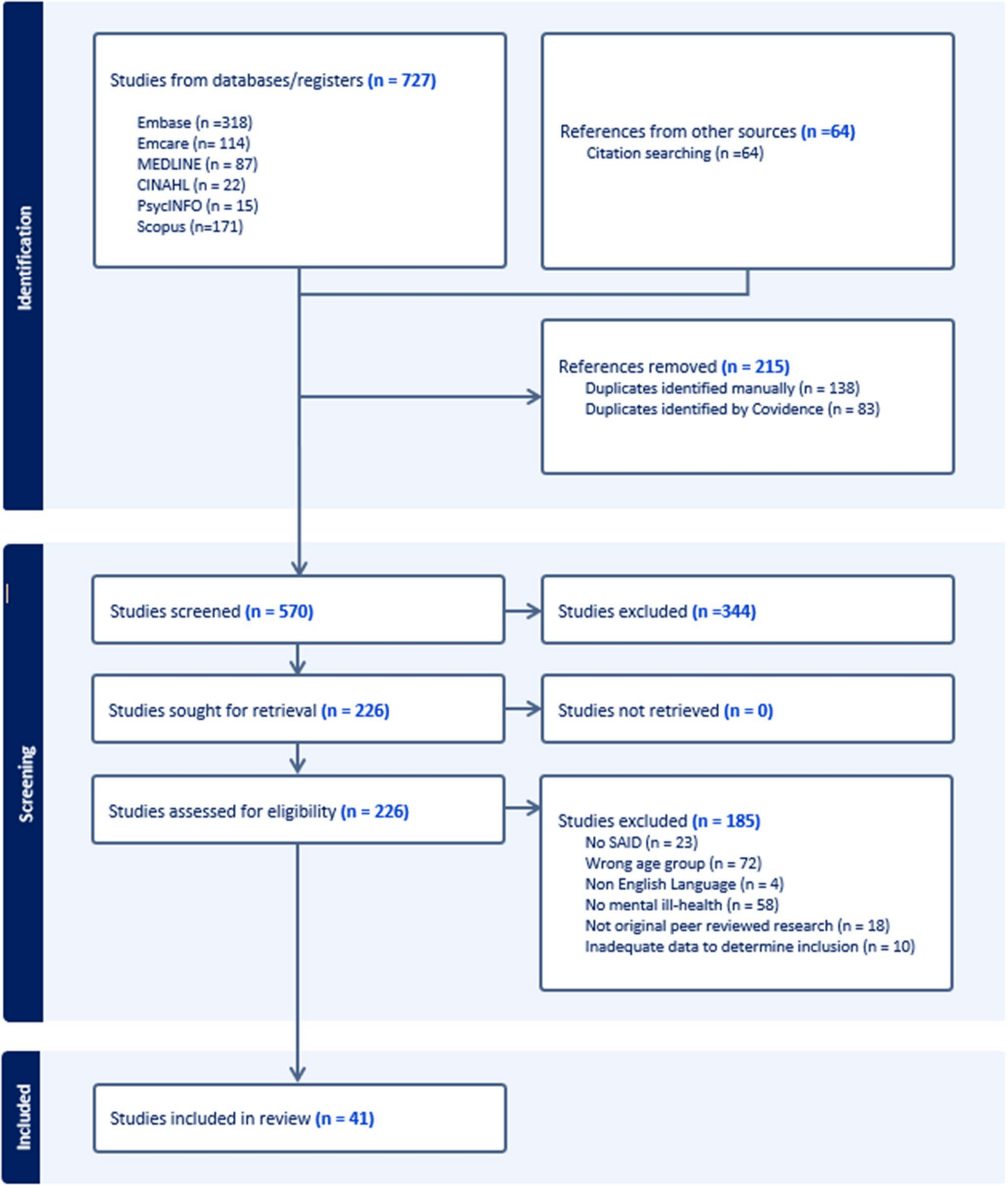
PRISMA flow diagram of screening and selection process of the scoping review

Titles, abstracts and citation information obtained from the database searches were exported to an Endnote library [49] and then to the Covidence review management system. The first author then screened all abstracts and titles for inclusion, selecting the full text of studies that met inclusion criteria. Where inclusion of an article was uncertain, this was reviewed by the second author and results discussed by the three authors until a consensus was achieved. All identification, screening and inclusion decisions were recorded in Covidence and published as a PRISMA flow diagram [46] (see Fig. 1).

The database search yielded a total of 727 articles and a further 64 articles were identified from citation searching. Following removal of 215 duplicates, 570 articles were screened. 344 articles were excluded as irrelevant, and 226 studies were retrieved for full text review. Of those, 185 were excluded. In total, 41 studies were included.

### Main outcome variables and analysis

Data extracted from the selected articles included: author/s, year of publication, country, sample size and age, study aim and type, SAID and mental ill-health diagnoses, SAID activity and mental health measures, and key findings related to mental ill-health. The first author extracted and coded data using a customised Microsoft Excel spreadsheet. The researcher first conducted a quantitative analysis and summary of the extracted data, including study location, type, and both SAID and mental ill-health diagnoses. A qualitative analysis followed, identifying patterns between the three study variables of mental ill-health, age, and SAID, cross referencing study, sample characteristics and study findings. Possible gaps in the research were thus identified.

## Results

### Study characteristics

The study characteristics and mental health related key findings of the included studies are listed in Table 1. Thirty nine of 41 studies were conducted in clinical settings, and 37 studies were observational by design. The most common research designs were case studies (*n* = 18), and cross-sectional studies (*n* = 14). Only one study, a Danish population-based retrospective cohort study by Zerwas et al. [7], investigated SAIDs collectively and was also the only to investigate eating disorders. Twenty-one studies were conducted in Türkiye and the remaining 19 came from 13 different countries, while one study was multinational, including multiple centres from 7 countries. Fourteen studies investigated Behçet’s syndrome and 15 FMF, reflecting the higher prevalence of these SAIDs, particularly in Türkiye, and their longer documented clinical histories [52, 53]. Other SAIDs studied were cryopyrin-associated periodic syndromes (CAPS) (2); chronic nonbacterial osteomyelitis (CNO) (2); mevalonate kinase deficiency (MKD) (1); tumour necrosis factor receptor-associated periodic syndrome (TRAPS) (3); systemic juvenile idiopathic arthritis (sJIA) (1); and periodic fever, aphthous stomatitis, pharyngitis, and adenitis (PFAPA) (1). Most studies (35) documented measures of disease activity using standard methods [37], and acute phase reactants (APR) were most cited (15). Other disease activity measures included SAID flare frequency per year (6), flare recency (1), and disease duration (7).

**Table 1:** Overview of studies included in the scoping review

Author(s); Year;	Country	Aims/purpose	Age: mean +/- SD; [median]; (range)	Youth sample size (Total sample size)	SAID	Study design	Mental health measure	Mental health-related key findings
Accorinti et al. [54]	Italy	To report on clinical course of ocular and extraocular involvement in patients with refractory Behçet’s syndrome.	22 years	1	Behçet’s syndrome	Case study	Medical assessment	Severe depression and suicidal ideation are adverse effects of SAID treatment Interferon α.
Akman-Demir et al. [55]	Türkiye	To determine whether Cyclosporine increases the risk of neurological involvement in Behçet’s with ophthalmology involvement.	28.3 years ± 8.1; [27 years]	269	Behçet’s syndrome	Retrospective cohort study	Medical assessment	Depression and neurological symptoms were more common in treatment group receiving only Cyclosporine compared to groups receiving other SAID treatments.
Alayli et al. [56]	Türkiye	To determine the prevalence of juvenile fibromyalgia syndrome (JFMS) in children with FMF and to evaluate quality of life (QOL) and depression.	12.21 years ± 2.74; (8–18 years).	91	Familial Mediterranean fever (FMF)	Comparative cross sectional	Children’s Depression Inventory (CDI);	Depression scores were higher for FMF participants than healthy controls. Chronic anxiety was significantly increased in FMF participants with JFMS compared to FMF without JFMS. FMF with JFMS had higher depression score (*p* = 0.007) and poorer QOL than FMF youth without JFMS.
Berody et al. [57]	France	To evaluate the patient’s medical referrals between the first symptom and the diagnosis of MKD and the diagnosis delay.	21 years; [20 years]; (12–29 years)	5 (13)	Mevalonate kinase deficiency (MKD)	Case series	Medical assessment	Of 13 patients, depression was present in 3, hyperactivity in one and lack of socialisation in another. Depression was more frequent in older patients with a longer gap between SAID onset and treatment.
Budman and Sarcevic [58]	United States of America	To report on a patient with Behçet’s syndrome, tic and obsessive-compulsive disorder.	22 years	1	Behçet’s syndrome	Case study	Medical assessment	Tic and obsessive-compulsive disorders were associated with neurological involvement in Behçet’s patient with symptoms including headache, cognitive decline, encephalopathy, dyskinesia and seizures.
Cetin et al. [59]	Türkiye	To investigate the depression and attack features in patients with colchicine resistant FMF.	28.2 years ± 9.3	100	FMF	Comparative cross sectional	Beck Depression Inventory (BDI)	Depression was more frequent in FMF patients with colchicine resistance, lower age of disease onset, longer latency in disease diagnosis and longer disease duration compared to non-colchicine resistant FMF patients.
Deger et al. [60]	Türkiye	To investigate the health-related quality of life (HRQoL) and mood conditions in familial Mediterranean fever (FMF) patients	[29 years]; (24–38 years)	157	FMF	Case control	Short form-36 (SF-36); Hospital anxiety depression scale (HADS)	Depression and anxiety were more frequent in FMF patients than in healthy controls. FMF patients have increased risk of anxiety, which may contribute to depression and mental ill-health. FMF was associated with lower QOL compared to healthy controls.
Deniz et al. [53]	Türkiye	To report on the first case of Neuro-Behçet’s Syndrome that presents with an acute psychotic attack.	18 years	1	Behçet’s syndrome	Case study	Medical assessment; Brief Psychiatric Rating Scale (BPRS)	Psychotic disorder due to SAID did not resolve with SAID treatment and partially resolved with psychiatric treatment. No family or personal psychiatric history, no psychosocial stressors. The psychotic episode was brief and occurred with SAID and neurological symptoms.
Durcan et al. [61]	Türkiye	To investigate the psychological symptoms, especially inattention/ hyperactivity of children and adolescents with FMF and to compare with healthy peers.	12.35 years ± 2.65; (8–18 years)	272	FMF	Case control	Strengths and difficulties questionnaire	SDQ scores indicating emotional and behavioural problems and inattention / hyperactivity were higher in FMF youth than in healthy controls. Inattention and hyperactivity scores were highest in FMF patients with disease onset less than 6 years. No significant correlation between SDQ total or subscale scores and patients’ age, age of onset, duration of ill-health, or SAID activity measures
Durcan et al. [62]	Türkiye	To compare sleep habits, depression and anxiety of patients with FMF to healthy children and to determine the influence of disease related factors on sleep habits and psychiatric symptoms.	12.4 years ± 2.6; (8–18 years)	323	FMF	Case control	Children’s Sleep Habits Questionnaire; Revised Child Anxiety and Depression Scale- Child Version (both validated Türkiye version)	Depression and anxiety were higher in FMF youth with sleep difficulties compared to those without. Sleep difficulties were more frequent in the FMF group. Those with a recent FMF flare had higher anxiety, depression and poorer sleep. No significant association between depression, anxiety or sleep and disease activity measures.
Düzçeker et al. [63]	Türkiye	To describe the relation between Quality of Life (QOL) and psychiatric symptoms in adolescents with systemic lupus erythematosus (SLE) and familial Mediterranean fever (FMF).	(13–18 years)	102	FMF	Comparative cross sectional	Brief Symptom Inventory (BSI); Simple Measurement of Impact of Lupus Erythematosus in Youngsters© (SMILEY©)- first question only.	Depression (28%) and negative self-view (12%) were less frequent among adolescents with FMF compared to healthy controls and participants with SLE. Psychopathology did not differ significantly between groups. FMF treatment non-responders were excluded from the study.
Fidan et al. [64]	Türkiye	Investigate the psychological effects of FMF on children and adolescents and their mothers.	12.2 years ± 2.9; (7–18 years)	50	FMF	Comparative cross sectional	Children’s depression inventory (CDI); State Trait Inventory for Children	No significant difference in depression or anxiety levels in youth with stable treated FMF compared to healthy controls.
Garcia-Delgar et al. [65]	Italy	To present the case of an adolescent with persistent tics and OCD with recurrent physical illness.	15 years	1	Tumour necrosis factor receptor associated periodic syndrome (TRAPS)	Case study	Medical assessment	Tics and OCD symptoms were episodic, worsening with SAID flare. Partial treatment efficacy with SSRI, corticosteroid and antipsychotic medication. Slow recovery with SAID biologic treatment.
Goldbach-Mansky et al. [66]	United States of America	To assess the safety and efficacy of rilonacept (IL-1 Trap), a long-acting IL-1 receptor fusion protein, in patients with a form of Cryopyrin-associated periodic syndrome (CAPS).	(20–64 years)	1 (5)	Cryopyrin associated periodic syndrome (CAPS)	Quasi experimental trial	Short form 36 (SF-36)	One participant with depression unrelated to SAID activity or treatment. Improved mental health outcome measures post-trial after first dose trial to three months and maintained at 2 years but overall scores not significant.
Gul et al. [67]	Türkiye	To evaluate the efficacy and safety of Canakinumab in adolescent and adult patients with FMF, who are resistant or intolerant to higher doses of colchicine.	[22 years]; (12–34 years)	13	FMF	Quasi experimental trial	Medical assessment	Anxiety an adverse effect of trial medication.
Hoffmann et al. [68]	Germany	To report on a patient with TRAPS who presented with neurological and psychiatric symptoms	16 years	1	TRAPS	Case study	Medical assessment	TRAPS can present with psychosis. Psychosis resolved with treatment of SAID with corticosteroid.
Houman et al. [69]	Tunisia	To analyse demographic, clinical and genetic features of Behçet’s syndrome in Tunisia and to compare them with other ethnic and geographic groups.	29 years; (6–58 years)	260	Behçet’s syndrome	Retrospective cohort study	Medical assessment	Psychiatric manifestation of Behçet’s syndrome occurred in 20.63% of study participants, including euphoria, loss of insight, disinhibition, indifference to the disease, and psychomotor impairment.
Hurst et al. [70]	New Zealand	To report on the first case of TRAPS associated psychosis.	13 years	1	TRAPS	Case study	Medical assessment	Adolescent presenting with recurrent inflammatory symptoms and organic psychosis. Once diagnosed effectively managed with SAID treatment.
Kacan et al. [71]	Türkiye	To examine the effects of individual education given to Turkish adolescents with FMF on anxiety, depression, and quality of life	14.6 years ± 1.82; (12–18 years)	70	FMF	Randomised control trial	State-Trait Anxiety Inventory for Children; Children’s Depression Inventory; Paediatric Quality of Life Inventory -Version 4.0	Anxiety and depression levels decreased in treatment compared to control group. The effect of treatment was significant only for depression.
Kisaarslan et al. [72]	Türkiye	To report on the treatment of four patients with colchicine overdose.	15 years	1 (4)	FMF	Case series	Medical assessment	Acute medical management of youth with FMF who attempted suicide by intentional overdose of colchicine.
Krasne et al. [73]	United States of America	To report on the diagnosis and treatment of an adult with PFAPA.	19 years	1	Periodic fever, aphthous stomatitis, pharyngitis, adenitis (PFAPA)	Case study	Medical assessment	Acute anxiety at hospital presentation of SAID flare. Anxiety resolved with SAID treatment.
Lai and Chan [74]	Taiwan	To report on the treatment of a case of refractory Behçet’s	23 years	1	Behçet’s syndrome	Case study	Medical assessment	Effective control of SAID with Interferon α2a but effects of treatment include severe depression.
Lee et al. [75]	United Kingdom	To report on the treatment of a complex case of a woman with Behçet’s.	27 years	1	Behçet’s syndrome	Case study	Medical assessment	Two episodes of post-puerperal psychosis and systemic inflammation. Diagnosed with Behçet’s and effectively managed with SAID treatment.
Lidor et al. [76]	Israel	To examine the association between FMF and depression and anxiety using big data analysis methodology.	27.4 years +/- 18.5 years	7670 (15340)	FMF	Prospective cohort study	Medical assessment	Anxiety and depression more prevalent in FMF group compared to controls (*p* < 0.001). Female gender, smoking and increasing age associated with depression and anxiety (*p* < 0.001).
Luzzati et al. [77]	Italy	To report on the diagnosis and management of a youth with SAPHO syndrome.	15 years	1	SAPHO syndrome	Case study	Medical assessment	Depression co-occurred and increased with other SAID symptoms. SAID symptoms resolved with SAID treatment, but depressive symptoms persisted.
Makay et al. [78]	Türkiye	To evaluate the depression and anxiety in paediatric FMF patients.	12.6 years ± 3.5 years. (7–12 years and 13–18 years)	47 (96)	FMF	Comparative cross sectional	Children’s Depression Inventory; Screen for Child Anxiety-related Emotional Disorders	FMF group depression scores higher than controls (*p* = 0.001). FMF and control group anxiety scores were not significantly different (24.8 ± 14 vs. 22.8 ± 12, *p* = 0.78).
Makay et al. [79]	Türkiye	To describe and compare the parent and child reported QOL of school-age children with FMF compared with healthy peers.	Youth participants 15.6 years ± 1.8	132	FMF	Comparative cross sectional	The Paediatric Quality of Life Inventory Version 4.0	FMF group emotional functioning QOL scores lower than healthy controls. FMF group had higher social function than healthy controls. QOL of FMF group was inversely correlated with the number of FMF flares, hospital presentations, and non-compliance with SAID treatment.
Mulders-Manders et al. [80]	Nether-lands	To quantify quality of life in patients with CAPS and assess the broader societal impact of CAPS on patient’s work and school.	[28.5 years]; (5–82 years)	8 (24)	CAPS	Cohort study	Euroqol EQ-5D-5 L; Child Health Questionnaire- Parent Form 50	86% of participants reported no mental health concerns and 14% of participants reported minor anxiety/depression. All participants were medically stable with treated SAID.
Onat et al. [81]	Türkiye	To investigate the effect of Selective Serotonin Reuptake Inhibitors (SSRI) on FMF flare frequency and acute response in patients resistant to SAID treatment, colchicine.	31 years +/- 10.3 years. (Youth participants 18–28 years)	6 (11)	FMF	Cohort study	Medical assessment	All participants experienced depression. Participants tolerated a higher colchicine dose when combined with an SSRI. SAID flare frequency and acute phase reactants significantly decreased after SSRI commenced (*p* < 0.001).
Oray et al. [82]	Türkiye	To report on a case of Behçet’s syndrome with therapeutic challenges.	20 years	1	Behçet’s syndrome	Case study	Medical assessment	Depression reported as adverse effect of Interferon.
Ozdemir et al. [83]	Türkiye	To report on a case of a youth with neuro Behçet’s presenting with psychosis.	29 years	1	Behçet’s syndrome	Case study	Medical assessment	Behçet’s syndrome with psychotic symptoms, Capgras syndrome, depression, anxiety, irritability, and neurological symptoms including headaches and white matter changes noted on MRI. Partial response to psychiatric medication improved when SAID treatment added.
Panicker et al. [84]	India	To report on a case of juvenile Behçet’s syndrome with strong family history to discuss the possible genetic basis of this disease.	12 years	1	Behçet’s syndrome	Case study	Medical assessment	Severe depression, deliberate self-harm, hostility and inattention, co-occurring with SAID symptoms. Authors notes strong family history of Behcet’s syndrome, severity increasing with subsequent generations.
Patel et al. [85]	United States of America	To report on a case of Behçet’s in a 17-year-old male presenting with acute psychosis.	17 years	1	Behçet’s syndrome	Case study	Medical assessment	Extreme mood disturbance, psychosis, aggression, inattention, insomnia, OCD, and neurological symptoms present. Psychotic symptoms resolved following SAID and psychiatric treatment, although memory, concentration and motivational problems persisted longer.
Pinto-Fernandez and Seoane-Reula [86]	Spain	To report on the case of Majeed Syndrome in a 13-year-old boy presenting with learning delays and attention deficit disorder.	13 years	1	Majeed syndrome	Case study	Medical assessment	Participant with history of attention deficit disorder and developmental delay, without further reference to mental ill-health, authors describe full recovery and ability “to lead a normal life” following successful SAID treatment.
Robertson and Ohta [87]	United States of America	To report on a case of Behçet’s in an 18-year-old male presenting with symptoms including depression, anxiety and substance use	18 years	1	Behçet’s syndrome	Case study	Medical assessment	Adult presenting with 4-year history of recurrent inflammatory symptoms which improved with SAID treatment. Management of mental health was not reported.
Ruperto et al. [88]	Multi-national	To determine dosing regimens for systemic JIA (sJIA) and polyarticular JIA (pJIA).	13.3 (3.2) years	26 (51)	systemic juvenile idiopathic arthritis	quasi experimental trial	Medical assessment	1 participant experienced psychiatric disorder (anxiety or depression) as an adverse effect of the SAID treatment.
Sezer et al. [89]	Türkiye	To evaluate differences in school performance and attendance, quality of life, and physical activity in adolescents with Familial Mediterranean fever (FMF) compared to healthy controls.	15.1 years +/- 2.7	129 (283)	FMF	Case control	Paediatric Quality of Life Inventory	Participants with FMF had lower self-reported QOL scores in psychosocial health domain compared to those in the control group (*P* = 0.028) and this difference was less significant than physical or school function domains. Differences in emotional and social function were not significantly different.
Sonmez et al. [90]	Türkiye	To investigate the associations between depression, anxiety and the health-related QOL of children with FMF compared with healthy peers.	12.6 years ± 2.58	130	FMF	Comparative cross sectional	Children’s Depression Inventory: Screen for Child Anxiety Related Emotional Disorders; Paediatric Quality of Life Inventory 4th Version	Higher anxiety with FMF group compared to healthy controls. Depression similar in both groups. Higher anxiety correlated with higher SAID severity. SAID duration was associated with depression. FMF group with high APR had higher anxiety than those with normal APR.
Taner et al. [91]	Türkiye	To evaluate depression and anxiety in participants with Behçet’s syndrome compared to psoriasis. To determine risk factors associated with depression. To compare psychologic distress of those with Behçet’s and psoriasis.	Total 33.18 years ± 7.31. Age stratified (18–25 years)	34 (207)	Behçet’s syndrome	Cohort study	Beck Depression Inventory (BDI) Beck Anxiety Inventory (BAI); Automatic Thoughts Questionnaire (ATQ), Beck Hopelessness Scale (BHS)	Depression risk 4 times higher for Behçet’s than psoriasis and 12 times higher for those with Behçet’s for more than 3 years. Anxiety scores higher in Behçet’s than psoriasis. Anxiety, not depression, was higher for young people with Behçet’s.
Yalçindag and Köse [92]	Türkiye	To compare the therapeutic effect of Infliximab and Interferon α-2a on refractory uveoretinitis in patients with Behcet’s syndrome.	26.8 ± 6 (range: 19–40)	53	Behçet’s syndrome	Retrospective cohort study	Medical assessment	Depression and insomnia were adverse effects in 7 (21%) participants treated with interferon. This was severe in 1 participant (3%) requiring discontinuation.
Zerwas et al. [7]	Denmark	To examine novel associations between autoinflammatory or auto immune diseases and associated family history and eating disorders.	(7–23 years)	3456 (966141)	SAIDs collectively	Retrospective cohort study	Medical Assessment	Young people with SAIDs have increased risk of developing eating disorders compared to controls. For eating disorders not otherwise specified risk ratio for SAID adolescent was 2.79 and for SAID male adolescent it was 8.40 times controls’ risk. Increased risk of developing eating disorders for adolescents with a SAID history in a first-degree relative.

### Mental illness in youth with SAIDs

Fourteen studies, examining FMF (12), Behçet’s (1) and SAIDs collectively (1), investigated the prevalence of mental ill-health in a SAID population using cross-sectional, case control or cohort designs. Eleven of these studies found mental ill-health was more prevalent in young people with SAIDs compared to healthy peers or peers with chronic diseases [7, 56, 59–62, 76, 78, 79, 90, 91]. Three studies found that measures of depression, anxiety and emotional and social functioning [63, 64, 89] were not significantly different in youth with stable treated FMF compared to healthy controls.

### Depression and anxiety in youth with SAIDs

Twenty-six of the 41 studies investigated depression and depressive symptoms. Alayli et al. [56] found that depression scores were higher in a sample of 91 adolescents with FMF compared to healthy controls regardless of disease severity. Cetin et al. [59] and Makay et al. [78] found that depression was significantly associated with SAID characteristics of treatment resistance, symptom severity, early onset, long treatment latency and long disease duration.

Anxiety and depression were both found to be associated with SAID activity in 13 studies. Lidor et al. [76] found strong associations between FMF, depression and anxiety (*p* < 0.001) in a large sample of 15,340 Israeli patients and matched controls. In their study of 43 paediatric patients with FMF compared to 53 healthy controls, Makay et al. [78] similarly found that increased depression and anxiety were significantly associated with increased incidence of SAID flares, hospital visits and SAID treatment non-compliance compared to healthy controls. In a study of 112 adults with Behçet’s with the median age of 33.18 years, Taner et al. [91] found that anxiety was significantly elevated in a subset of 16 young people aged 18–25 years compared to controls, and both depression and anxiety increased significantly with age and disease duration. Linear regression analysis by Taner et al. [91] found that a decrease in anxiety was associated with a decrease in depression. Sonmez et al. [90] found anxiety but not depression was associated with SAID severity and elevated APRs in their study of 130 adolescents with FMF. Similarly, Deger et al. [60] found that FMF was a risk factor for anxiety independent of the risk for depression, however the frequency of depression was dependent on concomitant anxiety.

Several paediatric studies [61, 62, 79] found that an increase in mental ill-health was associated with the recency of an acute SAID inflammatory flare or early age of SAID onset. In a comparative study of 323 children and adolescents with FMF and 260 healthy controls, Durcan et al. [62] found that those who had an FMF attack in the past month had considerably higher depression, anxiety, and sleep disturbance scores compared to controls. Most other comparative observational studies excluded participants with active or recent disease flares. Young people with FMF and sleeping issues had higher rates of depression and anxiety compared to young people without sleep issues and controls. Sleep disturbance has been shown to increase inflammatory levels [118], and vica versa [16]. Emotional and behavioural difficulties were also found to be more severe in SAID youth than in healthy controls [61, 79]. Makay et al. [79] found emotional functioning was inversely associated with the frequency of inflammatory flares in adolescents with treatment controlled FMF. Durcan et al. [61] found inattention and hyperactivity problems were significantly increased in youth with the onset of FMF before age six.

In summary, 29 studies indicated an association between SAIDs and mental ill-health, with depression and anxiety frequently identified. Eleven of 14 comparative studies found significantly higher scores on measures of mental ill-health in young people with SAIDs compared to controls.

### Eating disorders and SAIDs

A population-based prospective cohort study of approximately 1 million Danish children and adolescents investigated eating disorders and SAIDs collectively [7]. Zerwas et al. [7] found that the odds ratio of young people with autoinflammatory diseases, including predominately SAIDs, was 2.79 times higher for developing an unspecified eating disorder compared to their healthy peers. This ratio was 8.40 times higher for males with autoinflammatory diseases.

### Severe mental illness and severe SAID flares

Severe mental illness, including psychosis, severe depression, acute anxiety and acute onset obsessive compulsive and tic disorder, was primarily reported in case studies of severe SAIDs requiring acute medical management. Twelve of the 17 case studies diagnosed the presenting mental illness as organic or integral to the SAID inflammatory flare [53, 57, 58, 65, 68, 70, 73, 75, 77, 83–85].

### SAIDs with neurologic and psychiatric symptoms

Neurological manifestations of SAIDs are documented in the literature, including FMF, CAPS, MKD, TRAPS [36, 93] and Behçet’s [94]. Seven studies, involving Behçet’s [55, 58, 69, 83–85] and TRAPS [68], reported neurological symptoms, including headaches, facial palsy, hemiparesis, dyskinesia, seizures, abnormal brain imaging and mental ill-health including psychosis, depression, OCD and tic disorder.

### Mental health and quality of life measures

Mental ill-health was diagnosed by medical examination in 24 studies. Fifteen studies used 17 different standardised measures to quantify various mental health symptoms, with anxiety and depression measures most frequently used. Quality of life (QOL) was measured in nine studies [56, 60, 63, 71, 90], including 4 that used QOL measures only [66, 79, 80, 89]. These studies reported that the QOL and mental health of young people with SAIDs were significantly lower than healthy controls or negatively associated with SAID activity. Düzçeker et al. [63], using an unvalidated tool, found young people with stable treated FMF did not experience significant mental ill-health or reduced QOL compared to controls. Sonmez et al. [90], however, found lower QOL scores were associated with anxiety and SAID activity but not depression.

## Discussion

Thirty-one of the 41 studies included in this scoping review reported a clear association between mental ill-health and SAIDs in young people. Of particular note, 12 of these studies demonstrated consistent and significant findings, using large comparative samples. Eleven studies were cross-sectional, or case control studies, all sufficiently powered with large samples. These studies found that depression and/or anxiety scores were significantly higher in young people with SAIDs [56, 59–62, 76, 78, 79, 81, 89–91]. Depression was particularly associated with increased SAID severity, SAID treatment resistance, earlier onset, and/or longer treatment latency. Notably, one population-based prospective cohort study [7] also reported a significantly increased risk of developing eating disorders among young people with SAIDs, particularly for males, relative to peers without SAIDs. These findings are consistent with the considerable body of established literature on the role of inflammation in anxiety and depression [17, 18, 95, 96], and the high rates of depression among young people with immune system disorders [32, 97].

Depression and anxiety were also found to be significantly associated with SAID activity, including SAID flare frequency and recency [62], increased hospital visits, and SAID treatment non-compliance compared to healthy controls [79, 91]. Furthermore, increased SAID inflammatory serum markers, anxiety and depression were all associated with SAID earlier onset [61], longer SAID treatment latency, resistance [59, 81] and treatment non-compliance [78]. Systematic reviews and meta-analyses of inflammation in anxiety related disorders [98], depression [99] and ADHD [22] are consistent with these findings.

Anxiety and depression among young people with SAIDs increased in severity and prevalence with age [76, 90, 91]. Anxiety was found to be associated with inflammatory flares while depression was associated with anxiety [60, 90, 91], suggesting depression may be a marker of the cumulative impact of inflammation over time. These findings are consistent with the hypothesis by Slavich and Irwin [96] that inflammation mediates the temporal ordering of anxiety and depression. Lidor et al. [76], however, found that FMF was independently associated with both anxiety and depression. Disturbances of sleep, emotions and behaviour were also associated with SAID activity and early SAID onset [61, 62, 79], consistent with research identifying associations between learning delays, attention deficit/hyperactivity disorder [100–102], early childhood internalizing and behavioural problems among children with FMF [103].

### Severe SAID and mental illness

Severe mental illness and severe SAIDs, requiring acute care and limiting one or more life domains, were described in 10 of 16 case studies that investigated the medical management of SAIDs. The alignment of SAID flare and mental health symptoms at onset, progression, recovery or relapse [53, 65, 68, 70, 75, 83–85, 87] suggests an association that would benefit from further correlational and longitudinal research. Similarly, mental health symptoms only presented during flares of SAIDs in the case studies of young people with Behçet’s [58] and TRAPS [65] presenting with OCD and tic disorders. Furthermore, the observations of SAID activity and mental ill-health in case studies were largely consistent with the findings in studies involving other designs. For example, in their series of 13 cases with MKD, the observation by Berody et al. [57] that depression was associated with longer treatment latency was consistent with similar findings by Taner et al. [91] in their the cross-sectional study of young adults with Behçet’s.

### Limited association findings

Despite the above findings, it is important to recognise that associations reported between mental ill-health and SAIDs presentations was marginal or insignificant in several studies. While mental ill-health was referred to in two studies [66, 72], the authors did not further reference an association with the SAID studied. Goldbach-Mansky et al. [66] reported mental health outcomes were not significant in their quasi-experimental trial of rilonacept in CAPS patients, although the small sample size (*n* = 5) may have impacted this outcome. Seven studies reported that mental ill-health was an adverse effect of SAID treatment (particularly anxiety and/or depression), including four experimental and observational studies [55, 67, 88, 92]. Three were case studies of young people with refractory Behçet’s who reported depression following treatment [54, 74, 82], which may point to the established finding that immunomodulators can cause depression in various populations [104, 105].

Only two studies, investigating mental ill-health in youth with FMF, found no association between the variables. While the data provided by Fidan et al. [64] did not allow for further analysis, the study by Düzçeker et al. [63] of 26 stably treated young people with FMF excluded participants with chronic or recent SAID activity, which may explain their results.

Overall, the research suggests that SAID activity is associated with mental ill-health and that SAID treatment attenuates this association. Small sample sizes and an implicit assumption that identified mental ill-health is a secondary or adverse effect of SAID activity or treatment may have confounded findings.

### Reciprocal SAID and psychiatric treatment efficacy

The effect of anti-inflammatory and immune modulating treatments on mental ill-health has been extensively researched [106–109]. Case studies included in this scoping review frequently reported that psychiatric symptoms resolved following successful treatment of the SAID, with or without reference to psychiatric treatment [65, 68, 70, 73]. Furthermore, the included study by Onat et al. [81] reported that selective serotonin re-uptake inhibitors (SSRI) were associated with reduced SAID flare frequency and serum inflammatory markers. Similar results have been reported by other case studies of FMF patients [110, 111]. SSRIs have also been found to beneficially impact the disease progression of inflammatory bowel disease [97] and are being investigated as a disease modifying treatment in several studies of immune system disorders [112]. A meta-analysis of 45 studies has also provided strong evidence that conventional antidepressants have systemic anti-inflammatory effects [113].

Overall, the existing literature suggests that mental ill-health experienced by young people with SAIDs is associated with factors such as inflammatory flare features, disease duration and SAID treatment efficacy, and that SSRIs may be an important treatment option for young people with SAIDs who are at risk of mental ill-health.

### Study design

Most of the studies in this review that aimed to investigate mental ill-health in young people with SAIDs used observational designs, such as cross-sectional, case control or cohort studies, with large sample sizes. These studies generated a higher level of evidential weight than other represented study types (e.g., case studies) and produced consistent and significant findings supporting an association between SAID activity and mental ill-health. Large sample sizes were possible due to the high prevalence of Behçet’s and FMF in Türkiye [36, 52]. This, however, limits the generalisability of study findings to other SAIDs and locales. The large number of case studies, series and small open label trials reflect the rare disease status of SAIDs. Almost half of included studies were case studies, which are often excluded from reviews as poor-quality evidence. Sampayo-Cordero et al. [114] demonstrated that case studies can provide novel insights and approaches and contribute to the collective state of research and emerging understandings when included in reviews of rare diseases. Consistent with this, in this scoping review, case study findings provided further examples of identifiable patterns of mental ill-health and SAID activity, particularly in clinically severe and acute cases.

The presumption that mental ill-health was a secondary consequence of the adverse experiences of life with a SAID, rather than being integral to the disease, impacted some study design, results, and conclusions. The randomised control trial by Kacan et al. [71] examined the effects of education given to FMF youth on their anxiety and depression but did not measure SAID activity or examine its impact on participants’ mental health. This study, along with others [60, 63, 90, 91], excluded participants with prior psychiatric histories as confounding. This exclusion likely reduced the power of the results and limited possible insights.

### Implications for future research

Further reviews involving adults, young people and children with SAIDs are needed. This review identified research involving only ten SAIDs and only one study that investigated SAIDs collectively. There are, however, more than forty SAIDs [2] and at least 40% of diagnosed SAIDs are undifferentiated [36]. More sufficiently powered research is needed involving SAIDs not represented in this review; of SAIDs collectively, including undifferentiated types; and of SAID to SAID comparisons, to identify and distinguish patterns of mental ill-health within and across the SAID family. The high prevalence of FMF and Behçet’s in western Mediterranean countries, notably Türkiye, has enabled research with significant and compelling findings. This regional specificity, however, limits the generalisability of findings and more research in other locations is needed.

Studies investigating the health, including the mental health status, of medically treated and stable participants with SAIDs, and case studies of acutely unwell patients, were strongly represented in this review. Young people who were chronically unwell, with refractory SAIDs or with psychiatric histories, were excluded by some studies as confounding factors. Further research involving a wider spectrum of SAIDs and concurrent mental illness is needed. Systematic measurement of mental health before, during, and after SAID flares may further our understanding of the association between mental ill-health and SAID inflammatory flares, and help shed light on any causal relationships between them.

More prospective cohort studies and sufficiently powered case control studies are also required to investigate the risk and causal link between specific SAID activity and mental ill-health by including standard measures of different forms of SAID disease activity, inflammation, and mental health. Longitudinal designs are also required to discriminate between the risk of young people with SAIDs developing mental ill-health from the high background risk for mental ill-health among young people generally [10, 115, 116], not to mention the increased risk for mental ill-health associated with having a chronic disease [32].

## Summary & further implications

This scoping review is the first to explore the state of research regarding young people experiencing SAIDs and mental ill-health. The research describes a range of presentations of mental ill-health, from sub-clinical symptoms experienced by those with stable treated SAIDs, to severe acute presentations of SAIDs involving diagnoses of serious mental disorder. Most studies identified an association between SAID and mental ill-health in young people. Treatment resistance, pro-inflammatory markers, SAID severity, SAID flare recency and disease duration were particularly associated with anxiety or depression for specific SAIDs studied. Several studies of FMF and Behcet’s suggested a directional association between SAID inflammatory flare, anxiety, and depression. Links were also identified between early SAID onset and sleep, emotional and behavioural disturbances in FMF. Further research is required to determine whether these associations occur across other SAIDs.

This scoping review demonstrates a clear and consistent association between SAIDs, SAID activity and mental ill-health in young people with specific SAIDs. Mental health should be routinely assessed and treated in this population [117], and further research is required to inform clinical management guidelines. Research is also needed to generalise these findings to other age groups, locations and specific SAIDs, to quantify the risk to this population of developing mental ill-health, and to better understand the relationship between inflammation and mental ill-health.

## Electronic supplementary material

Below is the link to the electronic supplementary material.

Supplementary Material 1
