# Supplementary material for: Mental Ill-Health in young people with systemic autoinflammatory disease – a scoping review
Source: Rheumatol Int. 2025 Apr 18;45(5):108. doi: 10.1007/s00296-025-05864-w (PMC12008080; doi:10.1007/s00296-025-05864-w)
Supplement: Supplementary file 1 — Supplementary Material 1 [file 296_2025_5864_MOESM1_ESM.docx]

**Supplementary Material**

**Title:** Mental Ill-Health in Young People with Systemic Autoinflammatory Disease: A Scoping Review

**Journal name:** Rheumatology International

**Scoping review search terms per database**

| **Medline Database** |
| --- |
| Cryopyrin activation OR NLRP3-associated autoinflammatory disease OR Muckle Wells syndrome OR neonatal onset multisystem inflammatory disease OR chronic infantile neurological cutaneous and articular OR familial cold autoinflammatory syndrome OR LPIN2- chronic nonbacterial osteomyelitis OR Majeed syndrome OR Receptor antagonist deficiency OR Deficiency of the interleukin 1 receptor antagonist OR Deficiency of the interleukin-36 receptor antagonist OR NLRC4-associated autoinflammatory disease OR very-early-onset inflammatory bowel disease OR NLRP1-associated autoinflammatory disease OR NLRP12-associated autoinflammatory disease OR Periodic fever aphthous stomatitis pharyngitis and adenitis OR Behcet* syndrome OR Behcet* disease OR Schnitzler syndrome OR systemic juvenile idiopathic arthritis OR Type I interferonopathies OR Aicardi-Goutières syndromes OR monogenic systemic lupus erythematosus OR STING-associated vasculopathy, infantile-onset OR Singleton-Merten syndrome OR Type I IFN signalling OR Aicardi Goutières syndrome like autoinflammatory dis* OR X-linked reticulate pigmentary disorder OR SOCS1 haploinsufficiency OR Proteosome disorders OR proteosome associated autoinflammatory syndrome OR oligoadenylate synthetase deficiency OR spondyloenchondrodysplasia with immune dysregulation OR CEBPE associated autoinflammation) OR tumo* necrosis factor receptor-associated periodic syndrome OR ADAM17 deficiency OR neonatal-onset inflammatory bowel disease OR NOD2 associated granulomatous disease OR Blau syndrome OR early onset sarcoidosis OR deficiency of adenosine deaminase 2 OR rela haploinsufficiency OR Cleavage-resistant RIPK1-induced autoinflammatory syndrome OR CARD14 associated disease OR A20 haploinsufficiency OR LUBAC deficiency OR OTULIN-related autoinflammatory syndrome OR CDC42 deficiency OR ARPC1B deficiency OR Nck-associated protein 1-like deficiency OR PLCg2-associated antibody deficiency and immune dysregulation OR Cotamer associated protein-alpha syndrome OR vacuoles, E1 enzyme, X-inked, autoinflammatory, somatic syndrome OR SH3BP2 deficiency with multilocular cystic disease of the mandibles OR adaptor related protein complex 1 subunit sigma 3 deficiency OR laccase domain containing 1 deficiency OR sideroblastic anaemia with B cell immunodeficiency, periodic fevers and developmental delay OR systemic autoinflammatory dis* OR systemic autoinflammatory syndrome OR autoinflammatory dis* OR autoinflammatory syndrome OR periodic fever OR recurrent fever syndrome OR relapsing fever syndrome OR syndrome of undifferentiated recurrent fever OR undifferentiated systemic autoinflammatory dis* OR unspecified systemic autoinflammatory dis* OR undefined recurrent fevers hereditary recurrent fevers OR exp Mental Disorders OR anxiety or depression OR psychological distress OR psychological stress, limit to humans AND 1997 -Current AND adolescent 13 to 18 years OR young adult 19 to 24 years |

| **Emcare Database** |
| --- |
| IL-1 inflammasome disorders OR pyrin activation OR Familial Mediterranean Fever OR pyrin-associated autoinflammation with neutrophilic dermatosis OR mevalonate kinase deficiency OR  PSTPIP1 associated arthritis, pyoderma gangrenosum, acne syndrome OR Hyperzincemia and hypercalprotectinemia OR Periodic fevers with immunodeficiency and thrombocytopenia OR  Cryopyrin activation OR NLRP3-associated autoinflammatory disease OR Muckle Wells syndrome OR neonatal onset multisystem inflammatory disease OR chronic infantile neurological cutaneous and articular OR familial cold autoinflammatory syndrome OR LPIN2- chronic nonbacterial osteomyelitis OR Majeed syndrome OR Receptor antagonist deficiency OR Deficiency of the interleukin 1 receptor antagonist OR Deficiency of the interleukin-36 receptor antagonist OR NLRC4-associated autoinflammatory disease OR very-early-onset inflammatory bowel disease OR NLRP1-associated autoinflammatory disease OR NLRP12-associated autoinflammatory disease OR Periodic fever aphthous stomatitis pharyngitis and adenitis OR Behcet* syndrome OR Behcet* disease OR Schnitzler syndrome OR systemic juvenile idiopathic arthritis OR Type I interferonopathies OR Aicardi-Goutières syndromes OR monogenic systemic lupus erythematosus OR STING-associated vasculopathy, infantile-onset OR Singleton-Merten syndrome OR Type I IFN signalling OR Aicardi Goutières syndrome like autoinflammatory dis* OR X-linked reticulate pigmentary disorder SOCS1 haploinsufficiency OR Proteosome disorders OR proteosome associated autoinflammatory syndrome OR oligoadenylate synthetase deficiency OR spondyloenchondrodysplasia with immune dysregulation OR CEBPE associated autoinflammation OR tumo*ur necrosis factor receptor-associated periodic syndrome OR ADAM17 deficiency OR neonatal-onset inflammatory bowel disease OR NOD2 associated granulomatous disease OR Blau syndrome OR early onset sarcoidosis OR deficiency of adenosine deaminase 2 OR rela haploinsufficiency OR Cleavage-resistant RIPK1-induced autoinflammatory syndrome OR CARD14 associated disease OR A20 haploinsufficiency OR LUBAC deficiency OR OTULIN-related autoinflammatory syndrome OR CDC42 deficiency OR ARPC1B deficiency OR Nck-associated protein 1-like deficiency OR PLCg2-associated antibody deficiency and immune dysregulation OR Cotamer associated protein-alpha syndrome OR vacuoles, E1 enzyme, X-inked, autoinflammatory, somatic syndrome OR SH3BP2 deficiency with multilocular cystic disease of the mandibles OR adaptor related protein complex 1 subunit sigma 3 deficiency OR laccase domain containing 1 deficiency OR sideroblastic anaemia with B cell immunodeficiency, periodic fevers and developmental delay OR systemic autoinflammatory dis* OR systemic autoinflammatory syndrome OR autoinflammatory dis* OR autoinflammatory syndrome OR periodic fever OR recurrent fever syndrome OR relapsing fever syndrome OR syndrome of undifferentiated recurrent fever OR undifferentiated systemic autoinflammatory dis* OR unspecified systemic autoinflammatory dis* OR undefined recurrent fevers hereditary recurrent fevers AND Mental Disorders OR anxiety OR depression OR psychological distress OR Psychological Stress OR Psychological Distress OR Anxiety AND youth OR young adult OR emerging adult OR young people AND human AND 1997 - Current AND adolescent 13 to 17 years OR adult 18 to 64 years |

| **PsycINFO Database** |
| --- |
| IL-1 inflammasome disorders OR pyrin activation OR Familial Mediterranean Fever OR pyrin-associated autoinflammation with neutrophilic dermatosis OR mevalonate kinase deficiency OR PSTPIP1 associated arthritis, pyoderma gangrenosum, acne syndrome OR Hyperzincemia and hypercalprotectinemia OR Periodic fevers with immunodeficiency and thrombocytopenia OR  Cryopyrin activation OR NLRP3-associated autoinflammatory disease OR Muckle Wells syndrome OR neonatal onset multisystem inflammatory disease OR chronic infantile neurological cutaneous and articular OR familial cold autoinflammatory syndrome OR LPIN2- chronic nonbacterial osteomyelitis OR Majeed syndrome OR Receptor antagonist deficiency OR Deficiency of the interleukin 1 receptor antagonist OR Deficiency of the interleukin-36 receptor antagonist OR NLRC4-associated autoinflammatory disease OR very-early-onset inflammatory bowel disease OR NLRP1-associated autoinflammatory disease OR NLRP12-associated autoinflammatory disease OR Periodic fever aphthous stomatitis pharyngitis and adenitis OR Behcet* syndrome OR Behcet* disease OR Schnitzler syndrome.mp OR systemic juvenile idiopathic arthritis OR Type I interferonopathies OR Aicardi-Goutières syndromes OR monogenic systemic lupus erythematosus OR STING-associated vasculopathy, infantile-onset OR Singleton-Merten syndrome OR Type I IFN signalling OR Aicardi Goutières syndrome like autoinflammatory dis* OR X-linked reticulate pigmentary disorder OR SOCS1 haploinsufficiency OR Proteosome disorders OR proteosome associated autoinflammatory syndrome OR oligoadenylate synthetase deficiency OR spondyloenchondrodysplasia with immune dysregulation OR CEBPE associated autoinflammation OR tumo* necrosis factor receptor-associated periodic syndrome OR ADAM17 deficiency OR neonatal-onset inflammatory bowel disease OR NOD2 associated granulomatous disease OR Blau syndrome OR early onset sarcoidosis OR deficiency of adenosine deaminase 2 OR rela haploinsufficiency OR Cleavage-resistant RIPK1-induced autoinflammatory syndrome OR CARD14 associated disease OR A20 haploinsufficiency OR LUBAC deficiency OR OTULIN-related autoinflammatory syndrome OR CDC42 deficiency OR ARPC1B deficiency OR Nck-associated protein 1-like deficiency OR PLCg2-associated antibody deficiency and immune dysregulation OR  Cotamer associated protein-alpha syndrome OR vacuoles, E1 enzyme, X-inked, autoinflammatory, somatic syndrome OR SH3BP2 deficiency with multilocular cystic disease of the mandibles OR adaptor related protein complex 1 subunit sigma 3 deficiency OR laccase domain containing 1 deficiency OR "sideroblastic anaemia with B cell immunodeficiency, periodic fevers and developmental delay OR systemic autoinflammatory dis* OR systemic autoinflammatory syndrome OR autoinflammatory dis* OR autoinflammatory syndrome OR periodic fever OR recurrent fever syndrome OR relapsing fever syndrome OR syndrome of undifferentiated recurrent fever OR undifferentiated systemic autoinflammatory dis* OR unspecified systemic autoinflammatory dis* OR undefined recurrent fevers hereditary recurrent fevers OR Mental Disorders OR anxiety OR depression OR psychological distress OR Psychological stress OR Anxiety youth OR young adult OR emerging adult OR young people AND human AND 1997 -Current OR human AND adolescence age 13 to 17 years OR young adulthood age 18 to 29 years AND 1997 - Current |

| **Embase Database** |
| --- |
| IL-1 inflammasome disorders OR pyrin activation OR Familial Mediterranean Fever OR pyrin-associated autoinflammation with neutrophilic dermatosis OR mevalonate kinase deficiency OR PSTPIP1 associated arthritis, pyoderma gangrenosum, acne syndrome OR Hyperzincemia and hypercalprotectinemia OR Periodic fevers with immunodeficiency and thrombocytopenia OR Cryopyrin activation OR NLRP3-associated autoinflammatory disease OR Muckle Wells syndrome OR neonatal onset multisystem inflammatory disease OR "chronic infantile neurological cutaneous and articular" OR familial cold autoinflammatory syndrome OR LPIN2- chronic nonbacterial osteomyelitis OR Majeed syndrome OR Receptor antagonist deficiency OR Deficiency of the interleukin 1 receptor antagonist OR Deficiency of the interleukin-36 receptor antagonist OR NLRC4-associated autoinflammatory disease OR very-early-onset inflammatory bowel disease OR NLRP1-associated autoinflammatory disease OR NLRP12-associated autoinflammatory disease OR Periodic fever aphthous stomatitis pharyngitis and adenitis OR Behcet* syndrome OR Behcet* disease OR  Schnitzler syndrome OR systemic juvenile idiopathic arthritis OR Type I interferonopathies OR Aicardi-Goutières syndromes OR monogenic systemic lupus erythematosus OR STING-associated vasculopathy, infantile-onset OR Singleton-Merten syndrome OR Type I IFN signalling OR Aicardi Goutières syndrome like autoinflammatory dis* OR X-linked reticulate pigmentary disorder OR SOCS1 haploinsufficiency OR Proteosome disorders OR proteosome associated autoinflammatory syndrome OR oligoadenylate synthetase deficiency OR spondyloenchondrodysplasia with immune dysregulation OR CEBPE associated autoinflammation OR Tumo* necrosis factor receptor-associated periodic syndrome OR ADAM17 deficiency OR neonatal-onset inflammatory bowel disease OR NOD2 associated granulomatous disease OR Blau syndrome OR early onset sarcoidosis OR deficiency of adenosine deaminase 2 OR rela haploinsufficiency OR Cleavage-resistant RIPK1-induced autoinflammatory syndrome OR CARD14 associated disease OR A20 haploinsufficiency OR LUBAC deficiency OR OTULIN-related autoinflammatory syndrome OR CDC42 deficiency OR ARPC1B deficiency OR Nck-associated protein 1-like deficiency OR PLCg2-associated antibody deficiency and immune dysregulation OR Cotamer associated protein-alpha syndrome OR vacuoles, E1 enzyme, X-inked, autoinflammatory, somatic syndrome OR SH3BP2 deficiency with multilocular cystic disease of the mandibles OR adaptor related protein complex 1 subunit sigma 3 deficiency OR laccase domain containing 1 deficiency OR sideroblastic anaemia with B cell immunodeficiency, periodic fevers and developmental delay OR systemic autoinflammatory dis* OR systemic autoinflammatory syndrome OR autoinflammatory dis* OR autoinflammatory syndrome OR periodic fever OR recurrent fever syndrome OR relapsing fever syndrome OR syndrome of undifferentiated recurrent fever OR undifferentiated systemic autoinflammatory dis* OR unspecified systemic autoinflammatory dis* OR undefined recurrent fevers hereditary recurrent fevers AND Mental Disorders OR anxiety OR depression OR psychological distress OR Stress OR Psychological Distress OR Anxiety AND youth OR young adult OR emerging adult OR young people AND human AND 1997 – Current OR human AND 1997 - Current AND adolescent 13 to 17 years |

| **CINAHL Database** |
| --- |
| autoinflammatory dis* OR autoinflammatory syndrome OR periodic fever OR Cryopyrin Associated Periodic Syndrome* OR Muckle-Wells syndrome OR cryopyrin activation Tumo* Necrosis Factor Associated Periodic Syndrome OR Familial Mediterranean Fever OR Pyrin-associated Autoinflammatory Diseases OR Mevalonate Kinase Deficiency OR Hyperimmunoglobulin* D or Majeed syndrome OR Early-Onset Inflammatory Bowel Dis* OR Behcets dis* OR Behcets syndrome OR Behcets-like autoinflammat* OR Macrophage Activation Diseases" OR Familial Atypical Cold Urticaria OR Periodic fever, aphthous stomatitis, pharyngitis and adenitis OR Schnitzler syndrome OR Singleton-Merten syndrome OR Neonatal-onset inflammatory bowel disease OR Aicardi-Goutières syndromes OR Blau syndrome OR Rela haploinsufficiency OR A20 haploinsufficiency OR STING-associated vasculopathy, infantile-onset OR systemic-onset juvenile idiopathic arthritis OR syndromes of undifferentiated recurrent fever OR recurrent fever syndrome OR hereditary recurrent fever OR relapsing fever OR syndrome of undifferentiated recurrent fever OR undifferentiated systemic autoinflammatory dis* OR unspecified systemic autoinflammatory dis* OR undefined recurrent fevers OR hereditary recurrent fever AND mental disorders OR psychological distress OR anxiety OR depression OR psychological stress OR psychiatric illness OR mental illness AND adolescent 13-18 years and adult 19-44 years 2014-2022 OR adolescent 13-18years OR youth OR adolescent OR young people OR teen* OR young adult OR emerging adult |

| **Scopus Database** |
| --- |
| (TITLE-ABS-KEY("IL-1 inflammasome disorders" OR "pyrin activation" OR "familial Mediterranean fever" OR "pyrin-associated autoinflammation with neutrophilic dermatosis") OR TITLE-ABS-KEY("mevalonate kinase deficiency") OR TITLE-ABS-KEY("PSTPIP1 associated arthritis, pyoderma gangrenosum, acne syndrome") OR TITLE-ABS-KEY("Hyperzincemia and hypercalprotectinemia") OR TITLE-ABS-KEY("periodic fevers with immunodeficiency and thrombocytopenia") OR TITLE-ABS-KEY("Cryopyrin activation" OR "NLRP3-associated autoinflammatory disease" OR "Muckle Wells syndrome" OR "neonatal onset multisystem inflammatory disease" OR "chronic infantile neurological cutaneous and articular" OR "familial cold autoinflammatory syndrome") OR TITLE-ABS-KEY("LPIN2- chronic nonbacterial osteomyelitis OR Majeed syndrome") OR TITLE-ABS-KEY("Receptor antagonist deficiency OR Deficiency of the interleukin 1 receptor antagonist OR Deficiency of the interleukin-36 receptor antagonist") OR TITLE-ABS-KEY("NLRC4-associated autoinflammatory disease" OR "very-early-onset inflammatory bowel disease" OR "NLRP1-associated autoinflammatory disease" OR "NLRP12-associated autoinflammatory disease") OR TITLE-ABS-KEY("periodic fever aphthous stomatitis pharyngitis and adenitis") OR TITLE-ABS-KEY("Behcet’s syndrome" OR "Behcet’s disease") OR TITLE-ABS-KEY("Schnitzler syndrome") OR TITLE-ABS-KEY("systemic juvenile idiopathic arthritis") OR TITLE-ABS-KEY("Type I interferonopathies" OR "Aicardi-Goutières syndromes" OR "monogenic systemic lupus erythematosus") OR TITLE-ABS-KEY("STING-associated vasculopathy, infantile-onset OR Singleton-Merten syndrome") OR TITLE-ABS-KEY("Type 1 IFN signalling" OR "Aicardi-Goutières syndrome like autoinflammatory dis*" OR "X-linked reticulate pigmentary disorder" OR "SOCS1 haploinsufficiency") OR TITLE-ABS-KEY("Proteosome disorders" OR "proteosome associated autoinflammatory syndrome" OR "oligoadenylate synthetase deficiency" OR "spondyloenchondrodysplasia with immune dysregulation" OR "CEBPE associated autoinflammation") OR TITLE-ABS-KEY("tumo?r necrosis factor receptor-associated periodic syndrome" OR "ADAM17 deficiency" OR "neonatal-onset inflammatory bowel disease" OR "NOD2 associated granulomatous disease" OR "Blau syndrome" OR "early onset sarcoidosis" OR "deficiency of adenosine deaminase 2") OR TITLE-ABS-KEY("RELA haploinsufficiency" OR "Cleavage-resistant RIPK1-induced autoinflammatory syndrome" OR "CARD14 associated disease") OR TITLE-ABS-KEY("A20 haploinsufficiency" OR "LUBAC deficiency" OR "OTULIN-related autoinflammatory syndrome") OR TITLE-ABS-KEY("CDC42 deficiency OR ARPC1B deficiency" OR "Nck-associated protein 1-like deficiency" OR "PLCg2-associated antibody deficiency and immune dysregulation") OR TITLE-ABS-KEY("Cotamer associated protein-alpha syndrome" OR "vacuoles, E1 enzyme, X-inked, autoinflammatory, somatic syndrome" OR "SH3BP2 deficiency with multilocular cystic disease of the mandibles" OR "adaptor related protein complex 1 subunit sigma 3 deficiency" OR "laccase domain containing 1 deficiency" OR "sideroblastic anaemia with B cell immunodeficiency, periodic fevers and developmental delay") OR TITLE-ABS-KEY("systemic autoinflammatory dis*" OR "systemic autoinflammatory syndrome" OR "autoinflammatory dis*" OR "autoinflammatory syndrome" OR "periodic fever syndrome" OR "recurrent fever syndrome" OR "relapsing fever syndrome") OR TITLE-ABS-KEY("syndrome of undifferentiated recurrent fever" OR "undifferentiated systemic autoinflammatory dis*" OR "unspecified systemic autoinflammatory dis*" OR "undefined recurrent fevers hereditary recurrent fevers") AND TITLE-ABS-KEY("mental disorders" OR anxiety OR depression OR "psychological distress") AND TITLE-ABS-KEY(adolescent or "young adult" OR "young person" OR youth)) AND PUBYEAR > 1996 AND PUBYEAR < 2025 AND ( EXCLUDE ( SUBJAREA,"COMP" ) OR EXCLUDE ( SUBJAREA,"CENG" ) ) |
